# The acceptability of a novel seismocardiography device for measuring VO_2_ max in a workplace setting: a mixed methods approach

**DOI:** 10.1186/s12889-025-21480-6

**Published:** 2025-01-28

**Authors:** Anouska M Carter, Liam Humphreys, Alison Beswick, Sue Kesterton, Alex Bugg, Katharine Platts

**Affiliations:** Advanced Wellbeing Research Centre, Sheffield Hallam University, Sheffield, UK

**Keywords:** Seismocardiography, VO_2_ max, Cardiorespiratory fitness, Workplace health, Acceptability.

## Abstract

**Background:**

Workplace health screening rarely includes measures of cardiorespiratory fitness, despite it being a greater predictor of cardiovascular disease and all-cause mortality than other routinely measured risk factors. This study aimed to determine the comparative acceptability of using a novel seismocardiography device to measure cardiorespiratory fitness via VO_2_ max during a workplace health check.

**Methods:**

Participants were invited to participate in workplace health screening sessions where VO_2_ max was assessed by both seismocardiography at rest and sub-maximal exercise testing, in order for acceptability of both to be compared across multiple domains. Questionnaires and focus group guides for participants and practitioners were developed based on the Theoretical Framework of Acceptability. Data were analysed using t-tests and deductive thematic analysis.

**Results:**

There was a significant difference in the acceptability domain of ‘affective attitude’ between the novel SCG device (M = 9.06 ± 1.14) and the sub-maximal exercise testing (M = 7.94 ± 1.79); t = 3.296, *p* = .001, *d* = 0.50, and in the domain of ‘burden’ between the novel SCG device (M = 9.16, ± 0.55) and the sub-maximal exercise testing (M = 7.41 ± 1.45); t = 7.033, *p* = < 0.001, *d* = 1.45. Practitioners and employees highlighted the potential of seismocardiography to create a more inclusive and accessible workplace offer, allowing those with restricted mobility or those with differing physical or emotional needs to participate in wellness testing; yet there was a lack of understanding in both groups around intervention effectiveness and coherence.

**Conclusions:**

Seismocardiography may offer an acceptable route to cardiorespiratory fitness testing in the workplace, due to the low effort requirement and simplicity of administration. This study suggests that practitioners delivering such services have a critical role to play in acceptability of health interventions at work, as employees will be heavily influenced by practitioner beliefs around coherence and effectiveness. Comprehensive delivery training is important for the adoption of new health-related technologies such as seismocardiography into workplace health screening.

**Supplementary Information:**

The online version contains supplementary material available at 10.1186/s12889-025-21480-6.

## Background

An ageing population and an increase in the number of people living with chronic disease has led to a public health challenge, impacting quality of life, health care costs and workforce productivity [1]. Many chronic diseases such as heart disease and cancer are preventable by changes in lifestyle behaviours [2], with unhealthy lifestyles linked to poor health and decreased productivity at work [3, 4]. Workplace health interventions, such as diet and smoking education, exercise promotion, and environmental cues, are recognised as effective strategies for chronic disease prevention [5, 6]. Workplace health assessments are an important intervention to support early detection of health issues and are effective for reducing incidence of cancer [47] and diabetes [48], and for improving mental health outcomes of employees [49].

Workplace health assessments are traditionally part of the Occupational Health and overall workplace wellbeing offer. Typically, workplace health screening includes measurement of risk factors such as blood pressure and body mass index, but rarely includes measures of cardiorespiratory fitness (CRF), even though CRF is consistently reported as a greater predictor of cardiovascular disease and all-cause mortality than other routinely measured risk factors [7–12]. Poor CRF is associated with cancer risk [13] and carries greater mortality risk than any other cardiac risk factors, across all age groups, sexes, and races [14], with relatively small changes correlating well with mortality [15]. In turn, higher CRF is associated with better cognitive performance, particularly memory, executive function, and motor skills in middle-aged individuals [50], which may support improved performance at work.

Cardiorespiratory fitness refers to ‘the capacity of the circulatory and respiratory systems to supply oxygen to skeletal muscle mitochondria for energy production needed during physical activity’’ [16]. VO_2_ max (maximal oxygen consumption) is a standard predictive measure of CRF [17]. The gold-standard approach for assessing VO_2_ max is the cardiopulmonary exercise test (CPET) [20], which requires specialised equipment including a stationary bicycle and technical face mask that can monitor gas exchange, as well as sustained maximum effort from the participant and trained practitioners. Given the specialist nature of the CPET, and the time and effort required to undertake it, less burdensome sub-maximal exercise tests are commonly used, including the Astrand Rhyming Nomogram performed on a cycle ergometer [18] or the Chester Step Test [19], to predict V̇O_2_ max. Measurement via these methods takes less time, but is also less accurate than the CPET [20], and both alternative methods require equipment and modest effort, creating a barrier to adoption in workplace health screening.

The scientific statement from the American Heart Association recommends that all adults should have cardiorespiratory fitness measured during an annual health check [24], yet very few adults have access to this type of measurement at work, possibly due to constraints including lack of practitioner expertise and confidence or employee reluctance to exercise at work. Seismocardiography (SCG) offers an alternative option for assessing CRF from resting measurements in a few minutes, removing some of the potential barriers to fitness assessment in the workplace.

SCG is the recording of body vibrations induced by the heartbeat, providing information on cardiac mechanics, particularly heart sounds and cardiac output [21]. Using established prediction models, cardiac vibrations assessed via SCG can be used to calculate VO_2_ max [22]. Assessment of V̇O_2_ max using SCG is reported to be accurate for use in healthy populations (22) and is more accurate than other non-exercise prediction methods, such as algorithmic calculations using resting heart rate, for example (23). As such, it may be a beneficial addition to traditional health screening programmes, removing the need for costly and burdensome exercise testing. Little extant literature exists on the acceptability of fitness testing in the context of workplace health screening.

Acceptability is ‘*a multi-faceted construct that reflects the extent to which people delivering or receiving a healthcare intervention consider it to be appropriate*,* based on anticipated or experienced cognitive and emotional responses to the intervention*’ [25] and is a core component of feasibility testing [44]. Acceptability testing for workplace health interventions, as in clinical healthcare, has its philosophical roots in pragmatism, where focus on practical functioning and ‘actionable knowledge’ supports decision-making when financial resources are limited [26]. In recent years acceptability testing in relation to workplace health and wellbeing interventions has gained momentum, with acceptability measured alongside efficacy and feasibility for workplace interventions related to, for example, physical activity [27, 28], virtual reality [29], technology for mental health improvement [30, 31], food intake quality [32], and reduction in alcohol consumption [33]. Acceptability in this context is often measured via quantitative methods [51], yet some workplace-specific studies have sought to supplement quantitative findings with qualitative accounts of employee experience [52].

The aim of this study was to determine the acceptability of using a novel seismocardiography device to measure cardiorespiratory fitness during a workplace health check. The objectives of the study were:

1. To assess acceptability of CRF testing via novel seismocardiography and sub-maximal exercise to employees while at work, using quantitative and qualitative methods.
2. To assess acceptability of CRF testing via SCG and standard sub-maximal exercise to practitioners delivering health checks in a workplace setting, using qualitative methods.
3. To compare results across both methods of CRF testing to draw conclusions about which has greater acceptability.

The hypothesis was that the novel SCG device would be more acceptable than sub-maximal fitness testing, and thus may be more suitable for adoption into workplace health screening.

## Methods

### Study design & setting

A within subjects, mixed-methods study design was used, in order that any differences between the acceptability of CRF testing methods could be established, while also building an understanding of why one approach might be more acceptable than the other through the insight and experience of study participants and practitioners. This methodology is grounded in pragmatism as it relates to the implementation of health-improvement programmes in a workplace setting [26]. The study took place in two settings: first a public sector organisation (large UK university) and second a private sector organisation (medium-sized UK-based business) between August 2022 and February 2023. Approval was granted by the university Ethics Committee (ER45352278) in advance of study commencement, in line with the WMA Declaration of Helsinki ‘Ethical Principles for Medical Research Involving Human Participants’. All participants in the study provided written, informed consent to take part.

### Participant and practitioner recruitment

**Fig. 1:**
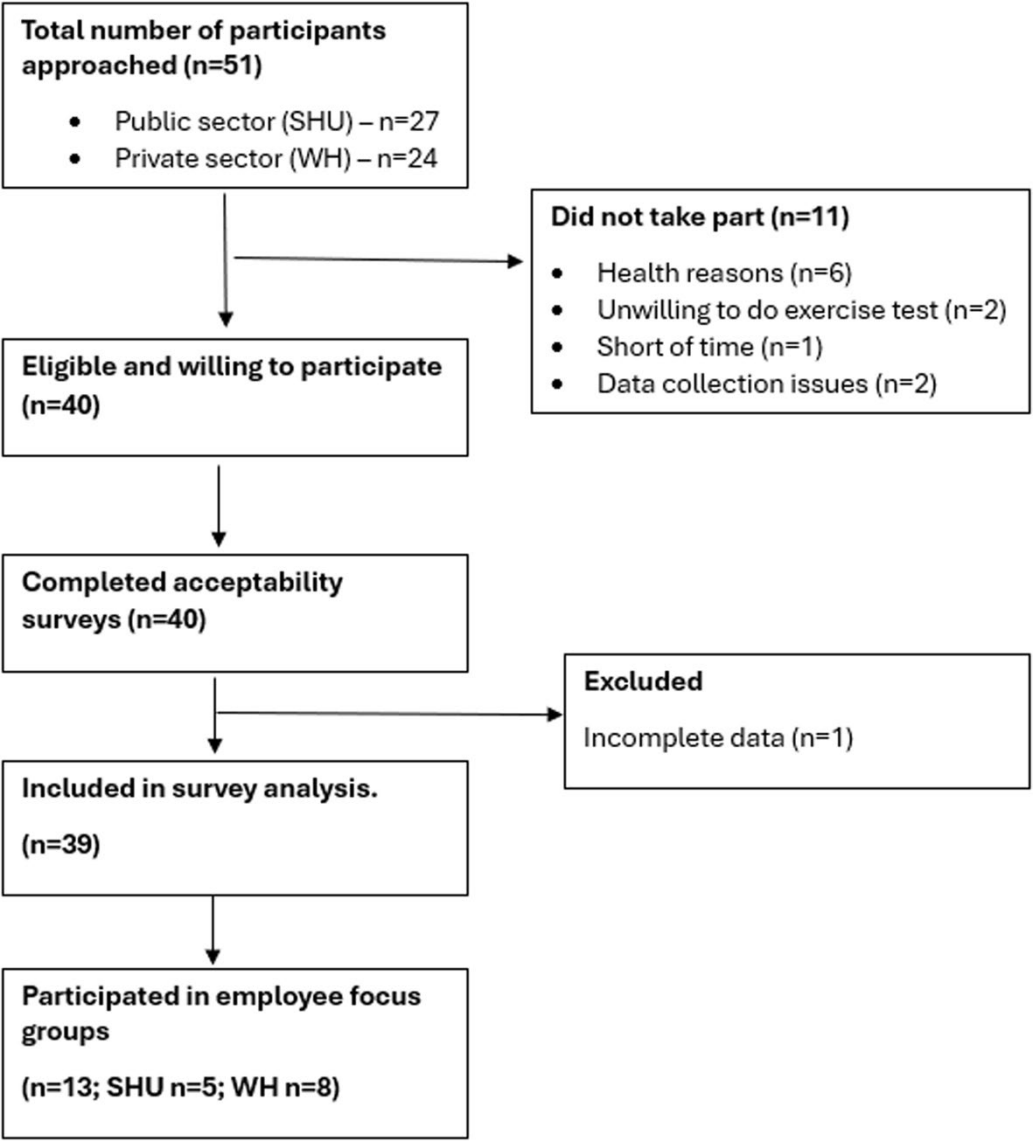
Participant recruitment flow chart

A convenience sample of employees was recruited via email invitation from individuals attending a workplace wellness appointment in the public (*n* = 20) and private (*n* = 20) sectors. Sample size calculations were not performed, with sample size based on sufficient participants for pilot or feasibility work, with the minimum recommended to be between 12 and 50 per group [34–37]. Participant inclusion and exclusion criteria are outlined in Table 1, while the participant recruitment process is outlined in Fig. 1. A purposive sample of six practitioners were selected to participate in this study, based on their experience of delivering the SHU Wellness programme in a university setting [39] or health checks in a corporate setting, and their understanding of CRF assessment methods.

**Table 1:** Participant inclusion and exclusion criteria

Inclusion Criteria	Exclusion Criteria
• Working in one of the organisations recruited from. • Accessing a health and lifestyle review at the organisations. • Aged over 18 years. • Able to provide written informed consent. • No electrical implant device. • No health complications that preclude them from safely participating in a sub-maximal exercise test.	• Failure to meet any of the inclusion criteria. • Electrical implant device fitted. • Medically advised not to undertake sub-maximal exercise or failure to meet pre-test screening criteria.

### VO_2_ max assessment via novel SCG

VO_2_ max assessment using the Ventriject Seismofit© [38] was added to an existing workplace health check and lifestyle review programme, “SHU Wellness”. The novel Ventriject Seismofit© SCG device is a small (50 × 25 × 15 mm), lightweight (16 g) plastic device which records vibrations caused by the heartbeat and transmits them to a cloud-based application via smartphone, which analyses them and calculates a predictive VO_2_ max value based on an established prediction model [22].

SHU Wellness is a one-hour screening session delivered in the workplace including testing of anthropometry (height, weight, body fat percentage, waist: hip), resting capillary bloods (total cholesterol, high-density lipoproteins, blood glucose), blood pressure (systolic and diastolic), resting heart rate and aerobic fitness (predicted V̇O_2_ max) using sub-maximal exercise testing (39).

Participants adhered to standard pre-test controls. They were requested to avoid food and caffeinated drinks for 2-hours, and refrain from alcohol and strenuous exercise for 24-hours, prior to their appointment. They were also requested to arrive well-rested and hydrated. Practitioners had no previous knowledge of the SCG device and were given a standard introduction to how it worked. Practitioners were aware of research regarding seismocardiography and V̇O_2_ max prediction but were not provided with specific information regarding the predictive power of SCG to participants. They were instructed to carry out both methods of CRF testing in order that the two methods could be compared for acceptability.

Upon arrival to the screening session, participants were asked to be seated and relax for five minutes in a chair, after which they were instructed to lie supine on a yoga mat on the floor. Practitioners demonstrated how and where the SCG device should be positioned on the chest, and participants were then instructed to attach the device themselves. The device used a hypoallergenic adhesive patch to adhere to the lower third of the sternum. This did not require clothing to be removed. If required, participants were requested to clean and shave a 5 cm patch of skin to ensure good contact.

The VO_2_ max measurement took approximately 30 s, during which time participants were asked to remain still and quiet, with arms relaxed by their sides. Once measurement was complete, the participant remained still for a further 2–3 min to enable the device to transmit results to the smartphone application, via an external server that provided the V̇O_2_ max calculation (this process was fully compliant with General Data Protection Regulations).

Once the VO_2_ max assessment using the SCG device was complete, participants undertook a sub-maximal exercise test using standard procedures for the Astrand-Rhyming Test [18, 40] for employees at the public sector site, and the Chester Step Test [19, 40] for employees at the private sector site. Both tests took approximately 10–15 min to complete and required moderate-intensity exercise by the participant.

### Data collection

#### Questionnaire

Acceptability scores related to both the SCG assessment and sub-maximal exercise testing were collected from employee participants immediately after the one-hour health wellness sessions via a custom questionnaire based on the Theoretical Framework of Acceptability for Healthcare Interventions (TFA) [25] and reviewed by a panel of experts, prior to the study. Questionnaires were administered online and consisted of 33 questions mapped to the seven TFA domains of ‘affective attitude, ‘burden’, ‘ethicality’, ‘perceived effectiveness’, ‘intervention coherence’, ‘self-efficacy’, and ‘opportunity costs’, with response options for each question along a 10-point Likert scale. High scores are desirable in all domains except burden, opportunity costs and ethicality, where lower scores are desirable. Table 2 outlines acceptability questions used in this study mapped against the TFA domains, with a description of each domain. Questionnaire in full is available in supplementary files.

**Table 2:** Acceptability questions mapped against the TFA [25, 41]

Theoretical Framework of Acceptability domain	Acceptability Questionnaire	
	SCG Device	Sub-maximal Exercise Test
**Affective attitude** *How an individual feels about an intervention* i.e., did you like or dislike, how comfortable did you feel, I’m pleased that I took part, I enjoyed.	Having my fitness test with Ventriject Seismofit was comfortable I liked having my fitness tested with Ventriject Seismofit Having my fitness tested with Ventriject Seismofit was enjoyable	Having my fitness test with a sub-maximal exercise test was comfortable I liked having my fitness tested with a sub-maximal exercise test Having my fitness tested with a sub-maximal exercise test was enjoyable
**Burden** *The perceived effort required to participate in the intervention.* i.e., how much effort did it take, has it been easy to find time, the length of the session was to long	I was able to tolerate having my fitness tested with Ventriject Seismofit Having my fitness tested with Ventriject Seismofit was convenient Having my fitness tested with Ventriject Seismofit took a long time I felt tired after having my fitness tested with Ventriject Seismofit	I was able to tolerate having my fitness tested with a sub-maximal exercise test Having my fitness tested with a sub-maximal exercise test was convenient Having my fitness tested with a sub-maximal exercise test took a long time I felt tired after having my fitness tested with a sub-maximal exercise test
**Ethicality** *The extent to which the intervention was a good fit for the individual’s value system.* i.e., there are morale and ethical consequences, it is fair to take part, fit well with how I want to live my life, it is important to me	I felt safe having my fitness tested with Ventriject Seismofit Having my fitness tested with Ventriject Seismofit was intrusive	I felt safe having my fitness tested with a sub-maximal exercise test Having my fitness tested with a sub-maximal exercise test was intrusive
**Intervention Coherence** *The extent to which a participant understands the intervention and how it works.* i.e. it is clear to me how xxx will help my xxx, I have received enough information	I value the information the Ventriject Seismofit fitness test gave me	I value the information the sub-maximal exercise test gave me
**Opportunity Costs** *The extent to which benefits*,* profits or values must be given up to engage in the intervention.* i.e., xxx interfered with my other priorities, I have altered my schedule to participate	I would like to have my fitness tested with Ventriject Seismofit as part of my routine healthcare check-ups	I would like to have my fitness tested with a sub-maximal exercise test as part of my routine healthcare check-ups?
**Perceived Effectiveness** *The extent to which the intervention is perceived as likely to achieve its purpose.* i.e., xxx has improved/helped xxx,	It was worth my while having my fitness tested with Ventriject Seismofit for the information that I received from the test I am confident that the information that the Ventriject Seismofit has given me about my health is accurate	It was worth my while having my fitness tested with a sub-maximal exercise test for the information that I received from the test I am confident that the information that the sub-maximal exercise test has given me about my health is accurate
**Self-Efficacy** *The participants confidence that they can perform the behaviour required to participate in the intervention.* i.e., how confident did you feel about xxx,	Having had my fitness tested with Ventriject Seismofit, I am confident I could conduct the Ventriject Seismofit test myself, with clear instructions, including applying the adhesive pad and navigating the phone app I feel confident I could complete my fitness assessment with Ventriject Seismofit: once a; week, month, 3-months, 6-months, year, never	I feel confident I could complete my fitness assessment with Ventriject Seismofit: once a week, month, 3-months, 6-months, year, never
**Other Comments**	If there is anything else you would like to tell us about having your fitness tested with Ventriject Seismofit, please write it in the space provided below	If there is anything else you would like to tell us about having your fitness tested with a sub-maximal exercise test, please write it in the space provided below

#### Focus groups

A convenience sample of employee participants took part in focus groups to explore views on the acceptability of the different methods of VO_2_ max prediction (focus group 1, public sector *n* = 5; private sector *n* = 8). 60-minute focus groups were delivered online via Zoom by an experienced qualitative researcher, shortly after the wellness sessions had completed. Focus group facilitation guides were developed to help facilitate the groups, which consisted of open-ended questions associated with the TFA domains, plus additional comments for further probing.

Practitioners who delivered the wellness sessions (focus group 2, *n* = 3), and practitioners who hadn’t delivered the sessions, but who worked in delivery of workplace health screening (focus group 3, *n* = 3), also took part in two 60-minute focus groups to assess prospective and retrospective acceptability from a practitioner perspective.

### Data analysis

#### Questionnaire data

Mean scores for each domain of acceptability for both the novel SCG device and the sub-maximal exercise test were calculated. For the three domains in which lower scores are desirable (burden, ethicality, and opportunity costs), scores were inverted to align positively with other domains and allow direct comparisons. Independent samples t-tests (equal variances) were performed on scores from each domain, as well as the overall acceptability score for each method of VO_2_ max testing, to ascertain significance of difference. Significance threshold was set at 0.05 (5%). Cohen’s *d* was calculated where significance was found, to determine effect size. Tests were performed using MS Excel for Windows 365 version.

#### Focus group data

Audio recordings from the focus groups were transcribed verbatim by a third-party transcription company. Deductive thematic analysis (TA) was performed using seven a priori themes reflecting the Theoretical Framework of Acceptability domains “affective attitude”, “burden”, “ethicality”, “perceived effectiveness”, “intervention coherence”, “self-efficacy”, and “opportunity costs”. Deductive TA was deemed most appropriate for this study, as it allowed analysis of the data against the Theoretical Framework of Acceptability and supported development of a robust answer to the research question [45,46]. Using this process, focus group data was coded against the seven thematic domains independently by two researchers (LH and KP) to reduce bias and enhance trustworthiness. Findings were then cross-referenced to refine, with final results verified by a third researcher (AC). Finally, the results from the questionnaires and the focus groups were compared to identify areas of congruence and difference, and to explain findings from an experiential perspective.

## Results

Fifty-one participants were invited to participate in the study. 11 people declined to take part as they were unwilling or unable to do the sub-maximal exercise test, leaving 40 study participants. 39 participants completed the acceptability questionnaire, and 13 participants joined the focus groups. Six practitioners joined a separate focus group.

Demographics are described in Table 3. Participants were predominantly female (60%), white British (87.5%), and middle-aged (42.4 ± 2.6), with an age range of 21 to 70. Participants were, on average, overweight (BMI > 25).

**Table 3:** Participant characteristics

	Total	Public Sector	Private Sector
**n**	40	20	20
**Sex (M**,** F)**	16 M, 24 F	7 M, 13 F	8 M, 12 F
**Age (years)**	42.4 ± 12.6	47.9 ± 11.8	37.0 ± 11.1
	21 to 70	24 to 70	21 to 57
**Ethnicity**	White British − 35	White British – 17	White British – 18
	Non-white British − 2	White non-British – 2	Non-white British − 1 Asian − 1
	White non-British − 2	Non-white British − 1	
	Asian − 1		
**BMI (kg/m**^**2**^**)** **Range**	25.7 ± 4.9 20.1 to 41.4	25.2 ± 4.3 20.1–39.0	26.1 ± 5.5 20.9 to 41.4

### Participant acceptability scores

There was a significant difference in the acceptability domain of ‘affective attitude’ between the novel SCG device (M = 9.06, SD = 1.14) and the sub-maximal exercise testing (M = 7.94, SD = 1.79); t = 3.296, *p* = .001, with significantly more positive sentiment towards the SCG assessment than the exercise test, and a medium effect size (Cohen’s *d* = 0.50). There was also a significant difference in the acceptability domain of ‘burden’ between the novel SCG device (M = 9.16, SD = 0.55) and the sub-maximal exercise testing (M = 7.41, SD = 1.45); t = 7.033, *p* = < 0.001, with the SCG assessment perceived as significantly less effort than the exercise testing, and a large effect size (Cohen’s *d* = 1.45).

In the other five domains, no significant differences between the groups were identified, although the mean acceptability scores were higher for the SCG device in all domains. Combining scores in all seven domains did identify a significant difference in overall acceptability between the novel SCG device (M = 9.24, SD = 1.15) and the sub-maximal exercise testing (M = 8.56, SD = 1.67); t = 5.558, *p* = < 0.001, with the SCG assessment considered significantly more acceptable overall, although the effect size was small (Cohen’s *d* = 0.33). Table 4 outlines acceptability scores and significance levels in all domains for compared methods of VO_2_ max testing.

**Table 4:** Acceptability scores for compared methods of VO_2_ max testing (*n* = 39)

	Ventriject Seismofit		Sub-maximal exercise					
	Mean	SD	Mean	SD	t	df	*p*	Cohen’s d**
**Affective attitude**	9.06	1.137	7.94	1.792	3.296	76	0.001*	0.50
**Burden**	9.16	0.552	7.41	1.453	7.033	76	< 0.001*	1.45
Ethicality	9.03	0.932	8.71	1.157	1.348	76	0.182	-
Intervention coherence	9.44	1.252	9.15	1.496	0.903	76	0.370	-
Perceived effectiveness	9.13	1.429	8.88	2.094	0.832	76	0.408	-
Opportunity costs	9.44	1.255	8.67	1.330	1.895	76	0.062	-
Self-efficacy	9.44	1.252	9.15	1.496	0.903	76	0.370	-
**Overall**	9.24	1.148	8.56	1.668	5.558	76	< 0 0.001*	0.33

*Significant where p=<.05

**Effect size where 0.2=small effect, 0.5=medium effect, 0.8=large effect

### Participant and practitioner experience

**Fig. 2:**
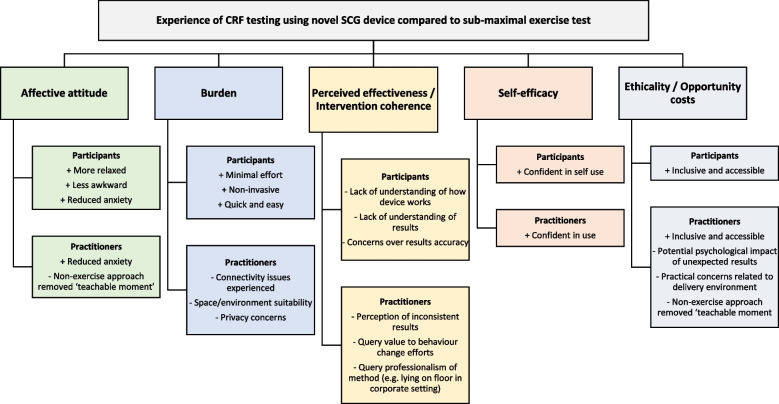
Participant and practitioner experience of CRF testing using the novel SCG device

Figure 2 illustrates the findings associated with each domain of acceptability grouped thematically from participant and practitioner focus groups.

#### Affective attitude

Employee feelings about SCG assessment via the novel device were positive overall, with users reporting that it felt less intimidating and less socially awkward than the sub-maximal test. Users reported feeling more relaxed with the SCG testing, and that it reduced the sense of anxiety and vulnerability commonly associated with the physical exercise test.

*“Easy to use*,* no exercise test necessary.” (Employee)*.

*“If someone’s not very comfortable with exercise*,* that sort of thing*,* whereas this*,* it’s literally just put this on*,* so it’s very easy to use…” (Employee)*.

*“…I felt really socially awkward doing the step test. I was like*,* please don’t judge me if I trip over anything!” (Employee)*.

*“[The practitioner] handled it really well when I had my SCG test*,* they explained it really clearly and just put me at my ease with it.” (Employee)*.

Both groups of practitioners had generally positive sentiment towards SCG assessment via the novel device, reporting that it had the potential to improve their consultations and provide more useful direction for goal setting and planning exercise intensity, as well as remove anxiety associated with exercise testing. However, neither group felt they fully understood the device. Practitioners who had delivered the SCG assessments discussed how including a physical exercise test in screening sessions had opened up discussions about physical activity, and how removing the physical test and replacing with an SCG assessment could potentially remove a discussion point and ‘teachable moment’ with clients.

*“They [employees] almost always say … I’m guessing you’re going to make me exercise*,* and some people*,* even though they are there*,* they do seem quite apprehensive about it.” (Practitioner)*.

*“I think actually doing some physical exertion can prompt more conversation about fitness and their feelings… which might then aid with the conversation about goal setting.” (Practitioner)*.

#### Burden

Employees considered the SCG assessment to require minimal effort and reflected that it was both non-invasive and did not require changing clothes or getting ‘hot and sweaty’ at work. Employees spoke about the burden of doing a physical exercise test at work, highlighting getting undressed/changed into workout clothes and physical exertion as significant barriers. In contrast, the SCG device was described as ‘quick and easy’ and would facilitate obtaining results regarding CRF.

*“People drop out because they don’t want to be having to get changed to do anything*,* they’ve got to get back to their day job.” (Employee)*.

There was a difference between the perception of burden between practitioners in focus groups 2 and 3. Practitioners in focus group 2, who had delivered assessments as part of the study, reported low to moderate burden retrospectively, and those issues causing effort had mainly been associated with repeated testing due to intermittent internet connectivity.

*“… You lose the result off the screen and then you’re like*,* right*,* can you do it again? And then you’ve got to wait five minutes for it to download again.” (Practitioner)*.

Practitioners in focus group 3, who had no direct experience of delivering SCG assessments but were professional workplace wellness coaches, prospectively perceived moderate to high burden. They voiced concerns with aspects of delivery in a corporate setting citing issues associated with space to lie down comfortably, asking people to position the device on their chest (mostly associated with clothing and privacy) and internet connectivity in host company locations. One practitioner mentioned the potential for an allergic reaction to the sticky patch used in the SCG assessment, although this was not reported or mentioned by any of the employee participants.

*“It’s the clothing issue*,* I think*,* for me. The fact that on a gent… the positioning’s right just on the sternum*,* but then thinking about a female doing that*,* if I was going in as a male member of staff delivering that*,* that might cause some kind of discomfort or make people feel a little bit apprehensive.” (Practitioner)*.

#### Perceived effectiveness and intervention coherence

Effectiveness as perceived by participants appeared to be heavily influenced by practitioner opinion. Employees generally said they did not understand the results from the SCG device or how they were generated, and, therefore, didn’t appreciate the significance of the results. As a result, the participants tended to trust the practitioner’s confidence in the accuracy of the readings. Additionally, there was a sense amongst participants that the exercise test felt more ‘authentic’ as a test of fitness, and that without physical exertion it was difficult to see how a CRF test via SCG device could work.

*“I asked which he [the practitioner] found to be closer*,* the reading from the device or from the step test and he said the device*,* so immediately I trusted that more.” (Employee)*.

*“Thinking of the bike test*,* one of the things that you get… is the immediate feedback about … whether I feel that I’m huffing and puffing more than I did last time.” (Employee)*.

*“The numbers [in the SCG assessment] didn’t mean anything to me and I was just focusing on the [exercise] test as being the proper test really*,* I suppose.” (Employee)*.

*“It would have added a bit more credibility to it if I understood how [the SCG device] was actually measuring that.” (Employee)*.

Practitioners in focus group 2 had mixed confidence in the accuracy of the results achieved. Some practitioners felt the device was more accurate than a sub-maximal test; however, others felt the numbers were inconsistent, making it challenging to explain results.

*“Without knowing exactly the accuracy of any of them relative to a full V̇O2 max test*,* it’s difficult to say*,* but if I had to*,* I would actually say I think the SCG device is more accurate.” (Practitioner)*.

*“Sometimes people’s Ventriject score was higher than the Step Test*,* sometimes it was lower*,* sometimes it was in a different category*,* sometimes it wasn’t*,* so it was quite confusing for the participants.” (Practitioner)*.

Practitioners in focus group 3 queried the value of results to daily life, for example, questioning whether CRF results would have sufficient meaning and relevance to the general population to drive health-related behaviour change. They also felt that asking people to lie down on the floor, which would likely be the case in a corporate setting, might compromise professionalism and cast doubt on the effectiveness of the assessment amongst clients.

#### Self-efficacy

Employees reported feeling more confident with the SCG device than with the exercise test, and said that for people with low physical confidence, the exercise test was a barrier. The simple administration and shorter duration of the SCG assessment also made people feel more confident about its use. Practitioners who delivered the assessment sessions using the SCG device reported feeling highly confident about its use, having had the opportunity to practice in advance with colleagues. There appeared to be no major issues with perceptions of capability or self-efficacy from either group.

#### Ethicality and opportunity costs

Both employee and practitioner groups reflected on how incorporation of a CRF assessment using the SCG device could create a more inclusive and accessible workplace offer, allowing those with restricted mobility, those screened out during risk stratification or those with differing physical or emotional needs to participate in wellness testing due to its non-invasive nature and low levels of sensory input. No negative ethical consequences were cited, and therefore no significant opportunity costs were identified in the employee participant group.

*“The device was great for me because I’ve got dodgy knees*,* so it was like on the step one*,* my knees were bad that day*,* so it like really*,* you know*,* I don’t think I was performing at my best*,* but whereas the device one was just like lying down.” (Employee)*.

*“I think in situations where it’s more sedentary employees*,* perhaps older adults*,* people with long-term health conditions that might not otherwise either be able to take part in the fitness assessment or be comfortable to do so.” (Practitioner)*.

Practitioners in focus group 2 voiced some concerns about the psychological impact of results on participants of multiple tests, reporting that people often ‘break down’ in wellness assessment sessions, and that there was an acute need for sensitivity, especially when there was a lack of understanding about results and in the case of vulnerable people.

For practitioners in group 3, who were giving a prospective view, the opportunity costs of delivery were more frequently associated with practical concerns, such as length of session, and having participants sufficiently engaged and relaxed for the assessment to be accurate, which is in line with delivery considerations in a corporate setting.

*“Nine times out of ten when I walk into a room to do my health checks*,* I’m either in a boardroom or I am in a little kind of cupboard somewhere*,* a little room off to the side somewhere*,* which is one that has chairs and tables in it. I think I’ve maybe had one or two occasions in the past 12 months where there’s actually a facility to have someone lay down on a bed*,* so I think if the test has to be done in that position*,* well*,* that’s an obvious barrier straightaway.” (Practitioner)*.

### Acceptability scores vs. qualitative feedback

Broadly, the experiences and perceptions of the employee participants as shared in the focus groups aligned with the scores from the acceptability questionnaires. The SCG device was found to be significantly more acceptable in the domains of ‘affective attitude’ (medium effect) and ‘burden’ (large effect) which was reflected in the comments relating to the amount of effort required to undertake the SCG assessment.

There were no significant findings related to perceived effectiveness or intervention coherence, which was strongly supported by statements related to a lack of understanding of how the device could be measuring fitness while no exercise was being undertaken. Similarly for ethicality and opportunity costs, no real ethical consequences of participating in the test were identified in the employee focus group, nor were any costs in terms of yielded benefits or values.

The overall acceptability score was significantly higher for the SCG device than for the sub-maximal exercise testing, but the effect size was small, which was borne out by the qualitative results, which appear far more balanced and not showing a clear preference towards one testing method or the other.

## Discussion

This study assessed VO_2_ max test acceptability amongst participants and practitioners assessing CRF using various predictive methods during a workplace wellness health screen. The findings broadly upheld the hypothesis that seismocardiography was a more acceptable method of workplace testing for cardiorespiratory fitness than sub-maximal exercise testing, but the evidence provided in this study was not strong in all areas of acceptability.

Focused at the individual level, the Theoretical Framework of Acceptability (TFA) provides an accessible yet comprehensive model with which to analyse data associated with a range of subjective experiences and emotions, using both quantitative and qualitative methods [25], which has been widely used in the healthcare context. Acceptability testing in the context of workplace wellness interventions is in its infancy, yet the recognition of acceptability as an important construct in this space is gaining ground [45]. Critically, acceptability testing using the TFA provides clear insights related to barriers and challenges that might hinder implementation and engagement of interventions, as demonstrated in this study. While employers seek to improve the wellbeing of their employees through workplace programmes, engagement with and adherence to programmes is likely to be higher when the experience is positive, enjoyable, well-understood and requiring low effort by those taking part.

The use of acceptability scores in multiple domains, supported by and compared to qualitative data, enabled an exploration of both positive factors and barriers to acceptability in the present study. In the domain of burden in particular, described as ‘*reasons for discontinuation and dropout’* in the TFA [25], the SCG device was assessed positively by employees, relieving them of the need to change clothes or to work up a sweat during the working day, removing a significant barrier to future engagement. Additionally, participants strongly liked the experience of using the device, which is a crucial acceptability factor in a workplace context where individuals may choose whether or not to engage.

Practitioners too were generally positive in terms of burden, articulated as low effort required, but place of delivery was an important factor. On a purely practical level, issues with device operation and Wi-Fi connectivity created an immediate barrier, and one that increased the effort of delivery for the practitioners, potentially creating opportunities for discontinued use of the SCG device in favour of alternative assessment methods. Practical and technical issues with technology have emerged as significant barriers to engagement in other studies of workplace wellbeing interventions [29]. Though it was not an explicit concern of participants in this study, the need to adhere a self-adhesive patch to clean, bare skin on the sternum could foreseeably create a considerable practical barrier in a corporate setting. This could be removed with the provision of comprehensive pre-test guidelines in advance, allowing participants to prepare their skin and clothing, if necessary. Consideration should also be given to potential confusion related to the constructs of self-efficacy and burden, which have been found in other studies using the TFA [53], which may confound results.

Although aspects of the SCG device were viewed positively, such as the low cognitive and physical burden required, opportunity costs were identified, as well as issues with intervention coherence and perceived effectiveness. For example, the ‘non-exercise’ approach to measurement of cardiorespiratory fitness is at odds with the primary goal of workplace health programmes, which is to promote healthy behaviours such as more movement [42]. Practitioners in this study discussed that removing the exercise component took away a potential discussion point or ‘teachable moment’, raising questions as to the ethicality of such an approach with a cohort engaged in health improvement activity. This could be mitigated by inclusion of advice and guidance related to physical activity or personalised health-related behaviour change support in any assessment using the SCG device.

There was a marked difference in the acceptability of the SCG device in the domain of perceived effectiveness between practitioner and employee groups, with the former appearing to rely on instinct in terms of the accuracy of the results, and the latter relying on the confidence and strength of the practitioner’s opinion. Where practitioners experienced conflicting results between the two methods of testing, there was confusion about the accuracy of the SCG device. Participant results may have been skewed by practitioner knowledge, which was not mitigated for in this study. This may explain why employee scores for perceived effectiveness and intervention coherence between the sub-maximal test and the SCG device were not significantly different and may have also affected participant scores for self-efficacy. Future studies should consider actions such as blinded assessments, where the participants have no knowledge of which device is providing their CRF results to reduce bias. Given the power of influence the practitioners had in this study, real-world delivery of such a screening programme would incur considerable costs for up-front practitioner training and education around the mechanism of action of the SCG device, accuracy of results, and the use of findings results in coaching conversations with individuals related to behaviour change.

In this study acceptability was tested as a standalone measure, albeit in two different workplace settings. To minimise the influence of poor perceived effectiveness on overall acceptability of workplace health interventions, the optimum approach may be to test effectiveness and acceptability in the same trial. For example, in testing immersive virtual reality for acceptability, wellbeing variables such as stress and relaxation have been measured alongside one another [29], while interventions to reduce workplace sitting have included physiological measures of blood glucose and lipids alongside general acceptability measures [28]. Where technology-based interventions for workplace fitness are assessed, there is clearly a need for user understanding and a belief in efficacy to support acceptability, and where that is not the case, high levels of attrition over time are observed [30].

The results in this study suggest potential benefits but highlight the need for a greater understanding of the barriers associated with SCG assessment in a workplace health context. In offering a low-effort, inclusive assessment method, there is an opportunity to reach a wider audience and support the efforts in health improvement for a larger cohort of employees, beyond those who are physically willing and able to participate in exercise testing.

### Limitations

This research project was a short-term acceptability study and was conducted on a small subset of two local workforces. The cohort lacked ethnic diversity, and the number of participants was not large enough to draw conclusions from different population subsets, such as by age or sex. Additionally, the study only recruited individuals who were able to undertake both types of testing method, so the views of those with limited mobility or contraindicators to exercise, for example, have not been captured. For these reasons, caution must be used in extrapolating the findings more widely. A lack of statistical power in this study reduces the external validity of the results, and while the reported participant experience broadly aligns with the quantitative results in this study, longitudinal studied with larger and more diverse cohorts would contribute a more profound understanding of the acceptability of the assessment method under study, and give greater transferability to a wider workforce population. Results may too have been confounded by the use of the two different types of sub-maximal exercise testing and the lack of understanding on the part of the practitioners of how the SCG device worked. Though based on the TFA [25], the questionnaire used in this study was not a validated tool, and, finally, a lack of previous acceptability data on CRF testing methods in the literature led to a limited discussion.

### Directions for future research

Future research is needed to explore broader implementation in the UK workforce and the potential impact on health and productivity from utilising the device on a larger scale. Potential settings include the workplace, where measurement of CRF is not currently offered, or within the NHS health check. A validated 8-item questionnaire based on the TFA is now available, which should considerably improve the comparability of acceptability research in the future, especially used in conjunction with qualitative methods [41, 43]. Further research to understand the role of practitioner training alongside the implementation of innovative health technologies is required, to better understand the influence of practitioners on user confidence and intervention coherence, for example. An exploration of costs associated with CRF testing in workplace health checks, via novel SCG and commonly used methods, as well as a Cost-Benefit Analysis would provide a richer understanding of acceptability, considering the position of both service providers who are delivering the health checks, and employers who make purchase decisions for their workforce .

### Conclusions and recommendations

This study assessed the acceptability of measuring CRF using various predictive methods to estimate VO_2_ max during a workplace wellness health check, providing evidence related to barriers that exist in the adoption of innovative technologies by both employees and practitioners. Our hypothesis that assessment via the SCG device would be significantly more acceptable for employees was correct, however the evidence was stronger in some domains of acceptability and weaker in others. Further studies are needed employing larger sample sizes and a broader range of participant mobility levels to determine acceptability across a broader spectrum of the UK workforce. Practitioners themselves have a critical role to play in acceptability of health interventions at work, as employees will be heavily influenced by practitioners’ beliefs around coherence and effectiveness of new technologies. With appropriate practitioner training seismocardiography offers a potential solution to increasing access to CRF in the workplace.

## Supplementary Information

Supplementary Material 1.

## Data Availability

The datasets used and/or analysed during the current study are available from the corresponding author upon request.

## Abbreviations

CRF: cardiorespiratory fitness
SCG: seismocardiography
TFA: Theoretical Framework of Acceptability
VO_2_ max: maximal oxygen consumption
