# Supplementary material for: The acceptability of a novel seismocardiography device for measuring VO2 max in a workplace setting: a mixed methods approach
Source: BMC Public Health. 2025 Jan 28;25:347. doi: 10.1186/s12889-025-21480-6 (PMC11773714; doi:10.1186/s12889-025-21480-6)
Supplement: Supplementary file 1 — Supplementary Material 1. [file 12889_2025_21480_MOESM1_ESM.pdf]

Your experience of VENTRIJECT SEISMOFIT fitness test - Questionnaire

Thank you for taking the time to read and complete this questionnaire. The purpose of this questionnaire is to help us understand what you think about having your fitness tested using the Ventriject Seismofit device.

Please read the statements below carefully. When you have read the statement, please rate how much you agree or disagree with it by circling a number. A score of 1 means that you strongly disagree with the statement. A score of 10 means that you strongly agree with the statement.

Participant ID: _________ Date: _________

1. **Having my fitness tested with Ventriject Seismofit was comfortable**

Strongly Disagree Neutral Strongly Agree

1 2 3 4 5 6 7 8 9 10

Comments____

2. **I was able to tolerate having my fitness tested with Ventriject Seismofit**

Strongly Disagree Neutral Strongly Agree

1 2 3 4 5 6 7 8 9 10

Comments____

3. **I felt safe having my fitness tested with Ventriject Seismofit**

Strongly Disagree Neutral Strongly Agree

1 2 3 4 5 6 7 8 9 10

Comments____

4. **Having my fitness tested with Ventriject Seismofit was intrusive**

Strongly Disagree Neutral Strongly Agree

1 2 3 4 5 6 7 8 9 10

Comments____

5. **Having my fitness tested with Ventriject Seismofit was convenient**

Strongly Disagree Neutral Strongly Agree

1 2 3 4 5 6 7 8 9 10

Comments____

6. **I value the information the Ventriject Seismofit fitness test gave me**

Strongly Disagree Neutral Strongly Agree

1 2 3 4 5 6 7 8 9 10

Comments____

7. **I would like to have my fitness tested with Ventriject Seismofit as part of my routine healthcare check-ups**

Strongly Disagree Neutral Strongly Agree

1 2 3 4 5 6 7 8 9 10

Comments____

8. **Having my fitness tested with Ventriject Seismofit took a long time**

Strongly Disagree Neutral Strongly Agree

1 2 3 4 5 6 7 8 9 10

Comments____

9. **It was worth my while having my fitness tested with Ventriject Seismofit for the information I received from the test**

Strongly Disagree Neutral Strongly Agree

1 2 3 4 5 6 7 8 9 10

Comments____

10. **I am confident that the information that Ventriject Seismofit has given me about my health is accurate**

Strongly Disagree Neutral Strongly Agree

1 2 3 4 5 6 7 8 9 10

Comments____

11. I liked having my fitness tested with Ventriject Seismofit

Strongly Disagree Neutral Strongly Agree

1 2 3 4 5 6 7 8 9 10

Comments____

12. Having my fitness tested with Ventriject Seismofit was enjoyable

Strongly Disagree Neutral Strongly Agree

1 2 3 4 5 6 7 8 9 10

Comments____

13. Having had my fitness tested with Ventriject Seismofit, I am confident I could conduct the Ventriject Seismofit test myself, with clear instructions, including applying the adhesive pad and navigating the phone app:

Strongly Disagree Neutral Strongly Agree

1 2 3 4 5 6 7 8 9 10

Comments____

14. I felt tired after having my fitness tested with Ventriject Seismofit

Strongly Disagree				Neutral				Strongly Agree	
1	2	3	4	5	6	7	8	9	10

Comments____

15. I feel confident I could complete my fitness assessment with Ventriject Seismofit:

Once per week	Yes ☐	No ☐
Once per month	Yes ☐	No ☐
Once every three months	Yes ☐	No ☐
Once every six months	Yes ☐	No ☐
Once a year	Yes ☐	No ☐
Never again	Yes ☐	No ☐

16. Would you pay for this test? ☐ Yes ☐ No

If yes, how much would you be willing to pay for the test? £________

17. If there is anything else you would like to tell us about having your fitness tested with Ventriject Seismofit, please write it in the space provided below:

End of questionnaire

Thank you for taking the time to complete this questionnaire. You participation in our research is appreciated.

Your experience of SUB-MAXIMAL EXERCISE TESTING - Questionnaire

Thank you for taking the time to read and complete this questionnaire. The purpose of this questionnaire is to help us understand what you think about having your fitness tested using a sub-maximal exercise test on a bike or step.

Please read the statements below carefully. When you have read the statement, please rate how much you agree or disagree with it by circling a number. A score of 1 means that you strongly disagree with the statement. A score of 10 means that you strongly agree with the statement.

Did you have your sub-maximal fitness test on a; Bike ☐ Step ☐

Participant ID: _________ Date: _________

1. **Having my fitness tested with a sub-maximal exercise test was comfortable**

Strongly Disagree Neutral Strongly Agree

1 2 3 4 5 6 7 8 9 10

Comments____

2. **I was able to tolerate having my fitness tested with a sub-maximal exercise test**

Strongly Disagree Neutral Strongly Agree

1 2 3 4 5 6 7 8 9 10

Comments____

3. **I felt safe having my fitness tested with a sub-maximal exercise test**

Strongly Disagree Neutral Strongly Agree

1 2 3 4 5 6 7 8 9 10

Comments____

4. **Having my fitness tested with a sub-maximal exercise test was intrusive**

Strongly Disagree Neutral Strongly Agree

1 2 3 4 5 6 7 8 9 10

Comments____

5. **Having my fitness tested with a sub-maximal exercise test was convenient**

Strongly Disagree Neutral Strongly Agree

1 2 3 4 5 6 7 8 9 10

Comments____

6. **I value the information the maximal sub-exercise test gave me**

Strongly Disagree Neutral Strongly Agree

1 2 3 4 5 6 7 8 9 10

Comments____

7. **I would like to have my fitness tested with a sub-maximal exercise test as part of my routine healthcare check-ups**

Strongly Disagree Neutral Strongly Agree

1 2 3 4 5 6 7 8 9 10

Comments____

8. **Having my fitness tested with a sub-maximal exercise test took a long time**

Strongly Disagree Neutral Strongly Agree

1 2 3 4 5 6 7 8 9 10

Comments____

9. **It was worth my while having my fitness tested with a sub-maximal exercise test for the information I received from the test**

Strongly Disagree Neutral Strongly Agree

1 2 3 4 5 6 7 8 9 10

Comments____

10. **I am confident that the information that the sub-maximal exercise test has given me about my health is accurate**

Strongly Disagree Neutral Strongly Agree

1 2 3 4 5 6 7 8 9 10

Comments____

11. **I liked having my fitness tested with a sub-maximal exercise test**

Strongly Disagree Neutral Strongly Agree

1 2 3 4 5 6 7 8 9 10

Comments____

12. **Having my fitness tested with a sub-maximal exercise test was enjoyable**

Strongly Disagree Neutral Strongly Agree

1 2 3 4 5 6 7 8 9 10

Comments____

13. **I felt tired after having my fitness tested with a sub-maximal exercise test**

Strongly Disagree Neutral Strongly Agree

1 2 3 4 5 6 7 8 9 10

Comments____

14. I feel confident I could complete a sub-maximal exercise test:

Once per week	Yes ☐	No ☐
Once per month	Yes ☐	No ☐
Once every three months	Yes ☐	No ☐
Once every six months	Yes ☐	No ☐
Once a year	Yes ☐	No ☐
Never	Yes ☐	No ☐

15. Would you pay for this test? ☐Yes ☐ No

If yes, how much would you be willing to pay for the test? £________

16. If there is anything else you would like to tell us about having your fitness tested with a sub-maximal exercise test, please write it in the space provided below:

Comments____

____

____

End of questionnaire

Thank you for taking the time to complete this questionnaire. You participation in our research is appreciated.
